# A comprehensive map of key aroma-active compounds in cigar tobacco via GC-IMS and GC-O-MS

**DOI:** 10.3389/fpls.2026.1749662

**Published:** 2026-03-18

**Authors:** Xue Wu, Shimin Liu, Zhongcheng Guo, Fengfeng Zhang, Tianze Liu, Yuqing Dou

**Affiliations:** 1 Tobacco Research Institute, Chinese Academy of Agricultural Sciences, Qingdao, China; 2 Graduate School of Chinese Academy of Agricultural Sciences, Beijing, China; 3 Shandong China Tobacco Industry Limited Company, Jinan, China

**Keywords:** aroma, cigar, flavor profile, gas chromatography-ion mobility spectrometry (GC-IMS), sensory evaluation

## Abstract

The aromatic characteristics of cigar tobacco leaves are the result of the release of aromatic compounds, but research on the distinctive aromatic profiles of cigars has been limited. This study employed GC–IMS combined with PCA to reveal differences in volatile components in cigar tobacco leaves. Additionally, HS-SPME and SBSE were used to extract volatile components from cigar tobacco leaves, which were then identified using GC–O–MS. Based on odor activity values, 15 out of 120 compounds were classified as aromatic active compounds. GC-IMS and GC-MS experiments indicated that “fatty” “grass” “fruity” “ammonia” “citrus” “chocolate odor” and “mint and camphor” were identified as characteristic aromatic attributes of cigar tobacco leaves. These characteristic aromas were associated with compounds such as Ammonia, 3-Methylbutanal, Pentanal, 2-Butanone, D-limonene, Nonanal, 6-Methyl-5-heptadiene-2-one, Geraniol and Neophytadiene. This study provides insights into improving cigar quality and promoting the development of new products with unique aromatic characteristics.

## 1 Introduction

Unlike traditional cigarettes, which contain a mixture of tobacco and other additives, cigars are a unique tobacco product made entirely from rolled tobacco leaves. They are characterized by their robust strength, rich tobacco flavor, and strong spicy (Shao et al., 2015; Wang et al., 2009), while having relatively lower levels of tar and nicotine. The international market has seen a steady rise in cigar sales (Qin et al., 2012), with the global cigar market now valued at $20 billion (Kowitt et al., 2023; Wu et al., 2023). Based on their functional roles, cigar tobacco leaves can be divided into two main parts: the filler and the wrapper, with the wrapper further subdivided into the binder and the outer wrapper. As the core raw material of cigars, the quality and aroma characteristics of the tobacco leaves directly determine the overall flavor profile of the cigar (Wirtz, 2012; Jin, 1982).

The aroma of cigars primarily stems from volatile compounds in the tobacco leaves, which not only impart distinctive flavors but also influence the sensory experience of consumers. In recent years, increasing attention has been paid to the types of volatile compounds in cigar tobacco leaves. These aroma compounds can be classified by their functional groups into acids, alcohols, esters, ketones, aldehydes, and heterocyclic compounds. Over 450 organic acids (Shen and Xian, 2003), more than 300 alcohols (Tang et al., 2007), over 500 esters (Zhao et al., 2019), more than 100 aldehydes, and over 500 ketones (Yan et al., 2020; Kunjapur et al., 2014; Ni et al., 2018) have been identified in tobacco. Additionally, tobacco contains various heterocyclic compounds such as pyrroles, furans, pyridines, pyrazines, indoles, quinolines, and carbazoles (Xu et al., 2014). The complex interplay of these volatile components forms the aromatic profile of cigar tobacco leaves. In 2013, Cigar Aficionado magazine first proposed eight major flavor categories for cigar tobacco leaves (Noble et al., 1984), including vegetal, spicy, floral, nutty, fruity, earthy, other distinctive flavors, and non-flavor characteristics. Building on this framework, ZHENGTF et al (Zhang et al., 2022). further refined the classification into subcategories such as nutty, beany, woody, peppery, fruity, caramel, honey, sweet, floral, herbal, milky, creamy, resinous, roasted, earthy, hay-like, leathery, sour, and powdery. Volatile organic compounds (VOCs) serve as the foundation of tobacco aroma, and their aromatic profiles play a crucial role in determining quality (Wampler, 2020; Starowicz, 2021).

The analysis of volatile compounds and aromatic profiles in cigar tobacco leaves can be conducted using techniques such as gas chromatography-ion mobility spectrometry (GC-IMS), gas chromatography-mass spectrometry (GC-MS), gas chromatography-olfactometry (GC-O), and electronic nose (e-nose) methods. GC-IMS is a volatile compound detection technology that combines the separation advantages of gas chromatography with the ability of ion mobility spectrometry to identify compounds based on differences in ion migration rates under an electric field (Wang S, et al., 2020; Gu et al., 2021; Liu et al., 2021). However, due to its nonlinear response, the IMS detector is less effective for precise quantitative analysis (Wang S. et al., 2020). Therefore, different sample pretreatment methods, along with GC-MS analysis, are still required for more accurate identification of volatile flavor compounds in various food and tobacco products (Chen et al., 2021). Common pretreatment methods for tobacco aroma analysis include simultaneous distillation extraction (SDE) and solid-phase microextraction (SPME). More recently, stir bar sorptive extraction (SBSE) has been introduced, though it remains less frequently reported. SPME is widely used in cigar tobacco aroma analysis due to its environmentally friendly nature and operational simplicity (Zhu et al., 2015; Qi et al., 2018). In contrast, SBSE replaces the SPME fiber with a stir bar, increasing the coating thickness while maintaining the same “like dissolves like” principle (Perestral et al., 2010). Compared to SPME, SBSE offers lower detection limits (Castro and Ross, 2015) and improves the accuracy of adsorbing semi-volatile compounds from aqueous solutions (Guerreroe et al., 2006), making it a promising technique for future applications.

Although existing research has revealed the contributions of certain volatile compounds to cigar aroma, a comprehensive understanding of the dominant aromatic profiles in cigar tobacco leaves remains incomplete. There is a lack of systematic studies integrating multiple analytical techniques to differentiate the characteristic aromas of cigars from those of traditional cigarettes. Therefore, this study employed HS-GC-IMS to evaluate volatile compounds in cigar tobacco leaves from different regions, clarifying variations and differences in their volatile compositions. Additionally, headspace solid-phase microextraction (HS-SPME) and stir bar sorptive extraction (SBSE) were used to extract and analyze aroma volatiles, identifying and quantifying key aroma-active compounds. GC-O and electronic nose techniques were also applied to characterize the primary aromatic profiles. The findings provide a theoretical foundation and practical guidance for developing the distinctive aromatic styles of cigars.

## 2 Materials and methods

### 2.1 Materials and equipment

Fourteen representative cigar tobacco leaf samples were selected from seven major production regions. All samples were provided by the Shandong Provincial Tobacco Administration, ensuring their varietal characteristics, curing, and aging conditions met industrial production standards. Detailed sample information is presented in **Table 1**. Sample Preparation and Storage: Upon arrival at the laboratory, all samples were immediately equilibrated for 72 hours in a constant temperature and humidity chamber (20 ± 2 °C, 60 ± 5% RH). Subsequently, leaves were destemmed and ground into powder using a specialized tobacco cyclone mill, then sieved through a 60-mesh screen. After thorough mixing, each sample was divided into three identical portions stored in brown glass bottles with sealing pads. Samples were kept in a -20 °C refrigerator under light-protected conditions until analysis to maximize stability of aromatic compounds. All chemical analyses were completed within two weeks of sample preparation.

**Table 1:** Information of cigar tobacco leaf samples.

Index	Production areas	Variety/type	Sample quantity	Harvest year	Processing methods	Aging period (months)	Key selection criteria
YN-pr	Yunnan Pu’er	‘Yunxue’ No. 1	1	2021	Air-drying	18-24	Typical Yunnan light-aroma style, fully matured
YN-mg	Baoshan Maguan, Yunnan	‘Yunxue’ No. 3	1	2021	Sun-drying	18-24	Rich aroma with pronounced sweetness
HB	Enshi, Hubei	‘Eyan’ Series	1	2021	Air-drying	12-18	Mellow aroma with ample intensity
NIA	Indonesia (East Java)	Besuki NO	1	Import Batch	Traditional Air-drying	>24	Imported primary ingredients, distinctive flavor profile, serving as international reference
HN	Danzhou, Hainan	‘Haixue’ Series	1	2021	Air-drying	12-18	Tropical climate characteristics, vivid aroma
SC	Panzhihua, Sichuan	‘Chuanyan’ Series	1	2021	Air-drying	12-18	Mountainous region characteristics, unique aroma
QL	Yishui, Shandong	‘Zhongxue’ No. 1	1	2021	Air-drying	6-12	Representative of northern production areas, serving as geographical and climatic reference

Normal ketones: 2-butanone, 2-pentanone, 2-hexanone, 2-heptanone, 2-octanone, and 2-nonanone (all analytical grade), Aladdin Company; C7-C30 n-alkanes purchased from Shanghai Sigma-Aldrich Trading Co., Ltd., 99.999% nitrogen; 20 mL headspace vials, Shandong Haineng Scientific Instrument Co., Ltd.; DB-5MS quartz capillary column (30 m × 0.25 mm, 0.25 μm), MXT-WAX capillary column (30 m × 0.53 mm, 1.0 μm), Restek Corporation, USA. Gas chromatography-olfactometry-mass spectrometry (GC-O-MS) system (Agilent 8890 GC + 7000D + Olfactometry Port), extraction head (120 μm DVB/CWR/PDMS), Agilent Technologies, USA. FlavourSpec^®^ gas ion mobility spectrometry, G.A.S. GmbH (Germany, Dortmund); CTC-PAL 3 static headspace autosampler, CTC Analytics AG (Switzerland, Zwingen); VOCal data processing software (0.4.03), G.A.S. (Germany, Dortmund).

### 2.2 Methods

#### 2.2.1 HS-SPME-GC-IMS

HS-SPME Analysis Conditions: After thoroughly mixing the sample, accurately weigh 1 g of the sample and place it in a 20 mL headspace vial. Add 10 µL of the internal standard 2-methyl-3-heptanone (100 ppm), incubate at 60 °C for 15 minutes, then inject the sample. Injection volume: 500 µL; no split injection; incubation speed: 500 rpm; Injection needle temperature: 85 °C; each sample is analyzed in triplicate.

GC Conditions: Column temperature: 60 °C; carrier gas: high-purity nitrogen (purity ≥ 99.999%); Programmed flow rate: initial flow rate of 2.00 mL/min maintained for 2 min, linearly increased to 10.00 mL/min over 8 min, linearly increased to 100.00 mL/min over 10 min, and maintained for 20 min. Chromatography runtime: 40 min; Injection port temperature: 80 °C.

IMS conditions: Ionization source: tritium source (³H); migration tube length: 53 mm; electric field strength: 500 V/cm; migration tube temperature: 45 °C; drift gas: high-purity nitrogen gas (purity ≥ 99.999%); flow rate: 150 mL/min; positive ion mode.

#### 2.2.2 HS-SPME/SBSE-GC-MS/O

HS-SPME Analysis Conditions: Thoroughly mix the sample. Accurately weigh 3 g of sample and 20 µL of internal standard 3-Hexanone-2,2,4,4-d4 (10 µg/mL) into a 20 mL headspace vial. Incubate at 60 °C for 15 min before injection. Three parallel replicates were analyzed per sample. Extraction was performed for 30 min using a 120-μm DVB/CAR/PDMS extraction head.

SBSE Analysis Conditions: Weigh 500 mg of sample into a 20 mL headspace vial. Add 20 μL internal standard 3-Hexanone-2,2,4,4-d4 (10 μg/mL) and 15 mL boiling water at 60 °C. Attach the PDMS magnetic stirring adsorption rotor, then seal the vial. Place the headspace vial in a 60 °C constant-temperature water bath magnetic stirrer. After 30 min of adsorption by the rotor, remove the vial. Clean residuals from the stirrer rotor surface with pure water, wipe dry, and immediately transfer to a gas chromatography vial for analysis. Place the gas chromatography vial containing the stirrer rotor in the thermal desorption unit to desorb the rotor material.

GC-MS analysis conditions: DB-5MS quartz capillary column (30 m × 0.25 mm, 0.25 μm); carrier gas: high-purity helium; column flow rate: 1.2 mL/min (constant flow mode); Injection port temperature: 25 °C; injection mode: splitless injection; injection volume: 2 μL; temperature program: start at 40 °C, hold for 3.5 minutes, then increase to 100 °C at 10 °C/min, then to 180 °C at 7 °C/min, and finally to 280 °C at 25 °C/min, holding for 5 minutes. Ion source: EI source; ionization voltage: 70 eV; ion source temperature: 230 °C; transmission line temperature: 280 °C; scan mode: full scan; scan range: 29–400 amu. Qualitative analysis was performed based on the total ion current chromatogram, peak retention time, spectral library (NIST 17 library), and retention index. Quantitative analysis was conducted using the internal standard hexane.

#### 2.2.3 GC-O

MS quadrupole temperature 150 °C, electron impact ion source, transmission line temperature 290 °C, electron energy 70 eV, scan range same as MS conditions. The split ratio between the odor detection port and the MS end is 1:1, odor detection port temperature 280 °C. GC-MS/O analysis was performed by five members who described the odor of the same sample to avoid subjectivity, recording odor characteristics and retention times.

Odor Activity Value (OVA) calculation: The concentration of key compounds is quantified by GC-MS, combined with the literature threshold (odor threshold), and OVA is calculated as concentration/threshold. An OVA >1 indicates a significant contribution to the fragrance.

#### 2.2.4 E-nose

Weigh 3 g of sample (such as tobacco dust or tobacco shreds), place the sample in a 20 mL headspace vial, seal it with a sealing membrane, and heat it at 60 °C for 30 minutes to fully release the volatile components in the sample. Place the pretreated sample in the sample chamber of the electronic nose. Start the electronic nose device to allow odor molecules in the sample chamber to enter the sensor array with the airflow. Sensors are sensitive to specific types of odor molecules, reacting with them physically or chemically to generate electrical signals. The electrical signals produced by the sensors are amplified, filtered, digitized, and transmitted to a computer. The computer uses algorithms to extract odor feature information. The extracted odor feature information is compared with a known odor database to identify the odor components in the sample, for details, please refer to **Table 2**.

**Table 2:** Chemical sensors and their corresponding sensitive substance types.

Number	Name of oxide sensor	Performance description
MOS1	W1C	Aromatic Compounds (Benzene Series)
MOS2	W5S	High sensitivity, particularly responsive to nitrogen oxides
MOS3	W3C	Sensitive to amine-based aromatic compounds(Ammonia)
MOS4	W6S	Primarily detect hydrides(Hydrogen)
MOS5	W5S	Alkane Aromatics (Short-Chain Alkanes)
MOS6	W1S	Sensitive to methane (methyl group)
MOS7	W1W	Sensitive to sulfides (inorganic sulfides)
MOS8	W2S	Sensitive to ethanol (alcohols)
MOS9	W2W	Aromatic component, sensitive to organic sulfides
MOS10	W3S	Sensitive to alkanes (long-chain alkanes)

### 2.3 Data processing

GC-IMS: Detect a mixture of six ketones, establish calibration curves for retention time and retention index, then calculate the retention index of the target compound based on its retention time. Use the built-in GC retention index (NIST 2020) database and IMS migration time database in the VOCal software to search and compare, thereby performing qualitative analysis of the target compound. Utilize plugins such as Reporter, Gallery Plot, and Dynamic PCA in the VOCal data processing software to generate three-dimensional spectra, two-dimensional spectra, difference spectra, fingerprint spectra, and PCA diagrams of volatile components, respectively, for comparing volatile organic compounds between samples.

GC-MS: Qualitative analysis of volatile substance components primarily relies on mass spectrometry standard libraries and retention index (RI) qualitative analysis. Retrieve the NIST standard library (NIST Chemistry WebBook, SRD 69) to identify the CAS numbers of the substance components. The retention indices recorded for the polar capillary column used in the experiment are compared with the calculated retention indices (RIs). If the error is less than 50, the retention is retained, indicating qualitative confirmation of the volatile components. The RIs of unknown compounds are calculated using the retention times obtained from n-alkanes C7 to C30 under the same gas chromatography-mass spectrometry (GC-MS) parameters.

Following the calculation method described in (Liu et al., 2021), the relative odor activity value (ROAV) is used to assess the contribution of each volatile component to the aroma of the cigar tobacco leaf sample.

All data were processed using SPSS 27 software. GC-MS data were primarily analyzed using the Maiwei Metabolism Cloud Platform, and Origin 2019 software was used to plot grid diagrams and correlation heat maps. Experimental results are expressed as mean ± standard error.

## 3 Results and discussion

### 3.1 Analysis of changes in volatiles in cigar tobacco leaves from different production areas using HS-GC-IMS

**Figure 1:**
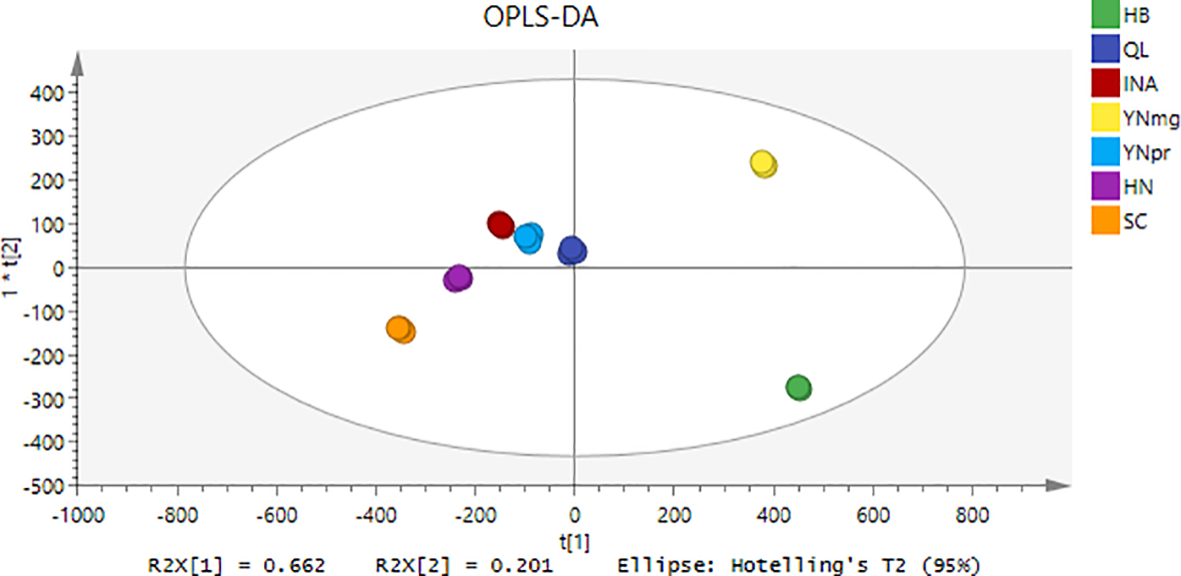
OPLS-DA diagram of cigar tobacco leaves from different production areas.

To investigate the differences in aroma components among cigars from different regions, a supervised pattern recognition method, Partial Least Squares-Discriminant Analysis (PLS-DA), was employed. The results are shown in **Figure 1**. As depicted in **Figure 1**, the seven groups of samples achieved significant separation, a result consistent with the classification model analysis conducted using the PCA model integrated into the FlavourSpec^®^ food flavor analyzer. The three key indicators for establishing the PLS-DA model—R²_X (proportion of X matrix information explained), R²_Y (proportion of Y matrix information explained), and Q² (model predictive capability)—were 0.662, 0.201, and 0.95, respectively, indicating that the model has good predictive capability and explanatory power for the data.

**Figure 2:**
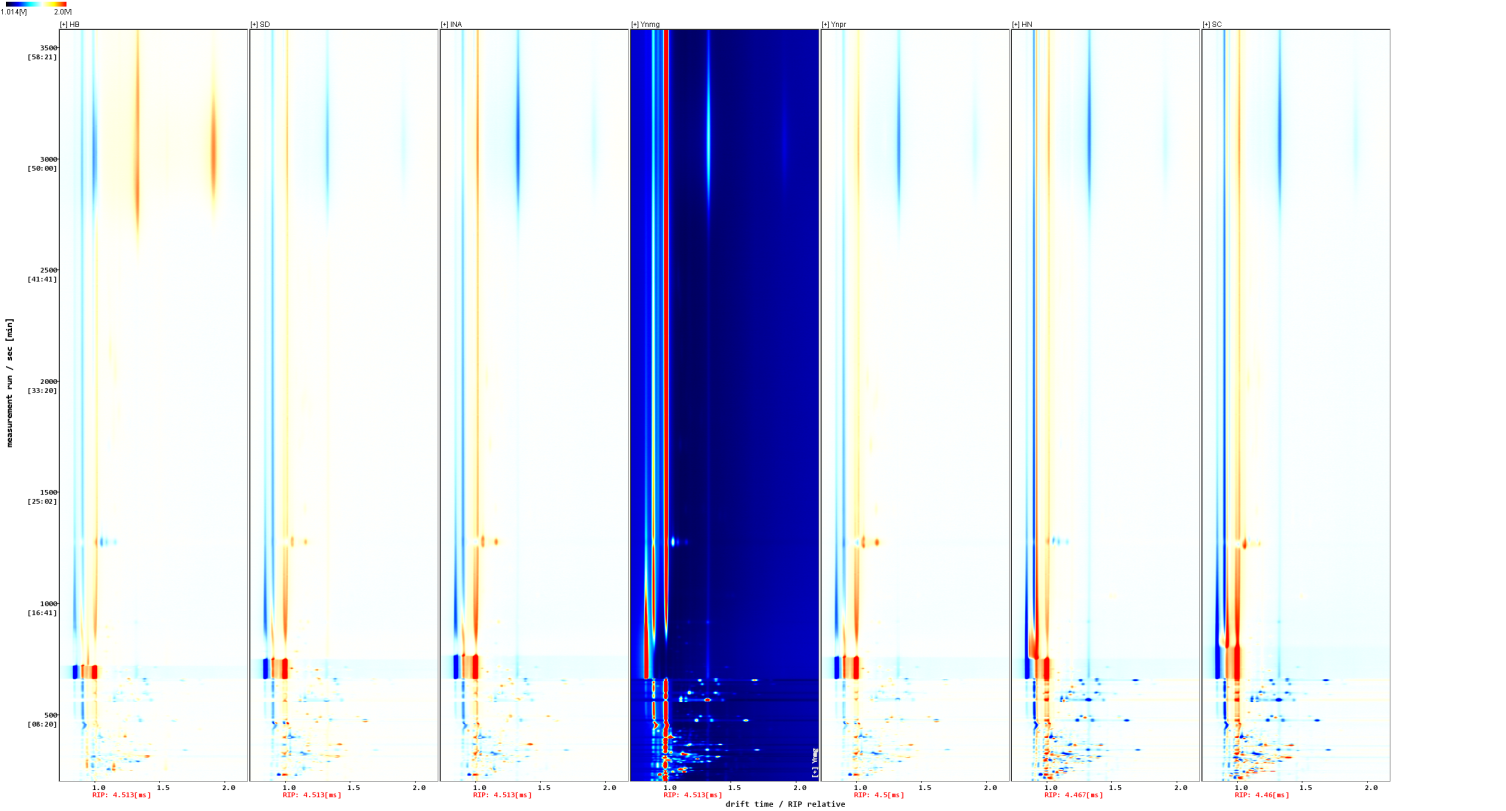
Two-dimensional GC-IMS spectrum of volatile components in the sample.

To visually compare the relative differences in volatile organic compounds among different samples, the GC-MS spectrum of the Yunnan Maguan sample (Ynmg) was used as the reference baseline. The relative difference spectra of each sample were obtained using the subtraction algorithm (Formula 1) to generate **Figure 2**. Based on normalization processing (peak area sum standardization), the color coding rules are as follows: white region (ΔI = 0): the target component concentration is consistent with the reference; red gradient (ΔI > 0): the target component concentration is significantly higher than the reference (color depth is positively correlated with log_2_(fold change)); blue gradient (ΔI < 0): the concentration of the target component is significantly lower than the reference (color intensity is negatively correlated with log_2_(fold change)). A significant number of red dots were observed in other smoke regions with retention times of 500–1000 seconds, indicating that the concentrations of these volatile compounds are higher in these regions compared to Ynmg (**Figure 2**). For the HN and SC smoke zones, red dots were also observed within retention times of 1000–1500 seconds. Additionally, a large number of blue dots were observed, indicating that the concentrations of these compounds were lower compared to Ynmg (**Figure 2**).

**Figure 3:**
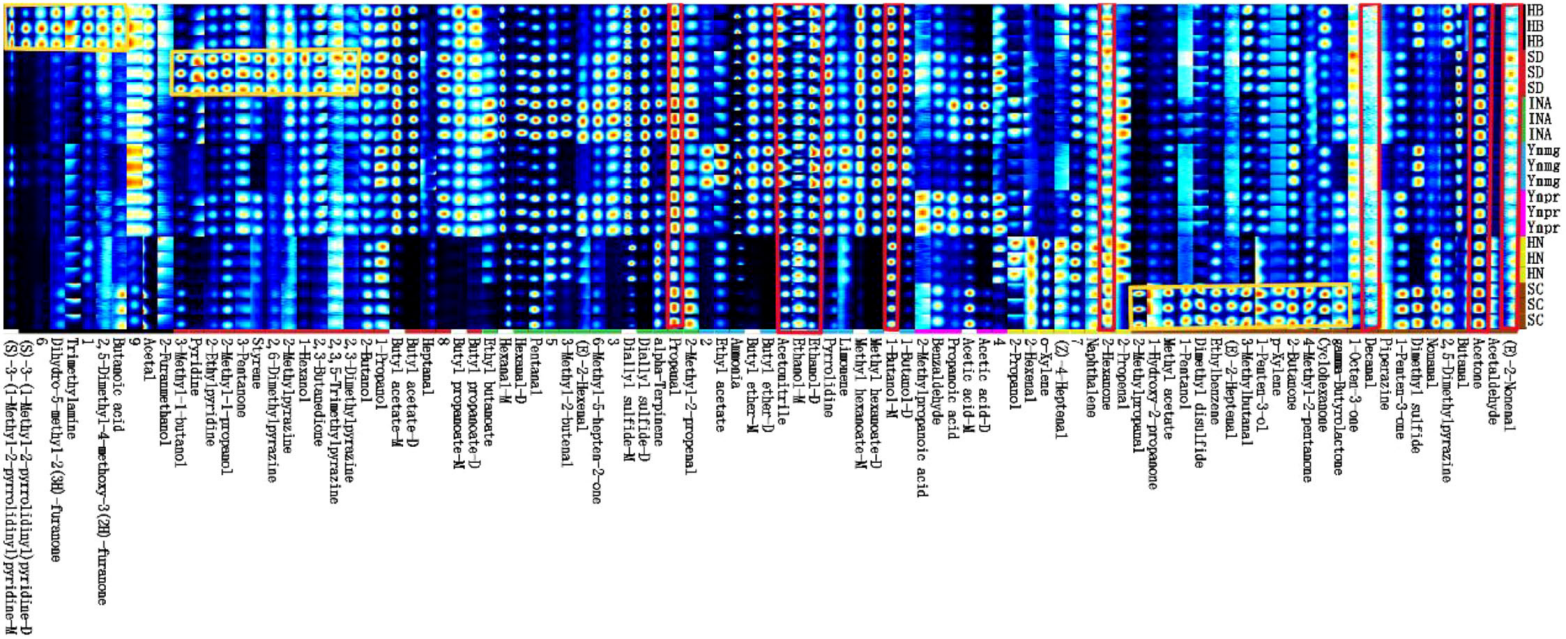
Fingerprint spectrum of volatile components in the sample.

Using GC-IMS technology to determine the volatile components in the samples, as shown in **Figure 3**, each row displays all the selected signal peaks in a single sample, and each column displays the signal peaks of the same volatile compounds in different samples. Brighter colors indicate higher concentrations, while darker colors indicate lower concentrations. A total of 78 volatile compounds were detected in the cigar tobacco leaf samples, including 16 aldehydes, 13 alkaloids, 11 alcohols, 11 ketones, 8 esters, 5 aromatic hydrocarbons, 4 acids, 2 terpenes, 3 sulfides, 2 ethers, and 3 unidentified compounds. The number of detected compounds was more diverse than reported in previous studies. The compounds highlighted in red are those present at high concentrations in all tested samples, including (E)-2-Hexenal (trans-2-hexenal), acetonitrile, decanal, 2-hexanone, 1-butanol-M, ethanol-M, ethanol-D, acetaldehyde, and propanal (Shi et al., 2023). These compounds, marked with yellow rectangles (**Figure 3**), may be unique aromatic characteristic substances of cigar tobacco leaves from various smoking regions. This study found that although there are some differences from previous studies on aromatic components, ketones, aldehydes, and alcohols are the main aromatic components in cigar tobacco leaves (Wang et al., 2014; Shi et al., 2006). The volatility of ethanol aids in the diffusion of aromatic molecules, particularly highlighting the “clear and crisp” characteristic in light-aroma baijiu. Previous studies have shown that aldehydes such as propionaldehyde and acetaldehyde possess pleasant fruity, fresh, and nutty roasted aromas (Brunton et al., 2000), while decanal is positively correlated with citrus-woody aromas in cigar tobacco leaves (Zheng, 2022). Aldehydes such as acetaldehyde, propionaldehyde, and decanal are primarily produced through amino acid degradation and microbial conversion (Kunjapur et al., 2014). In the Maillard reaction, amino acids undergo deamination and decarboxylation, leading to the formation of aldehydes (Wang et al., 2019). Amino acid degradation, microbial conversion, and the Maillard reaction are influenced by reaction time, temperature, and humidity (Kunjapur et al., 2014; Wang et al., 2019).

The VIP values for each variable xk of the volatile components in the sample were calculated by summing the squares of the PLS weight coefficients wak. When the Variable Importance in Projection (VIP) value exceeds 1, it indicates that the variable makes a significant contribution to the overall model. As shown in **Table 3**, VIP > 1 and P ≤ 0.05: these variables are deemed important. The important substances include Ammonia, (S)-3-(1-Methyl-2-pyrrolidinyl)pyridine-M, (S)-3-(1-Methyl-2-pyrrolidinyl)pyridine-D, Acetic acid-M, Diallyl sulfide-D, Dimethyl sulfide, Acetic acid-D, 3-Methylbutanal, Pentanal, and 2-Butanone. The primary aroma profiles include ammonia-like odor (ammoniacal), strong pungent odor, garlic odor (garlic), cabbage, sulfur, gasoline (cabbage, sulfur, gasoline), spicy flavor (spicy), chocolate, fatty flavor (chocolate, fat), green grassy odor with a faint banana scent (green grassy, faint banana, pungent), fruity aroma, and camphor scent (fruity, camphor). The main aromatic compounds extracted from cigar tobacco leaves include 10 types, but there are differences compared to the high-content aromatic components mentioned above. They exhibit a pattern where compounds with smaller aromatic contributions have higher concentrations, while those with larger aromatic contributions have lower concentrations. The reason for this may be that compounds such as alcohols, aldehydes, ketones, and esters may interact through synergistic or additive effects (Zhong et al., 2022).

**Table 3:** Identification of aroma-active components in cigar tobacco leaves based on SIMCA VIP values (GC-IMS method).

Compounds	OD^A^	VIP	Probability						
			QL	INA	YNmg	YNpr	HN	SC	HB
Ammonia	Ammoniacal	4.93	0.00002	0.000	0.000	0.000	0.000	0.000	0.000
(S)-3-(1-Methyl-2-pyrrolidinyl)pyridine-M	Strong pungent, Woody	4.34	0.004	0.0012	0.013	0.002	0.0013	0.0014	0.000
(S)-3-(1-Methyl-2-pyrrolidinyl)pyridine-D	Strong pungent, Woody	2.90	0.020	0.017	0.027	0.018	0.017	0.0175	0.015
Acetic acid-M	Spicy sour	1.88	0.000	0.000	0.000	0.000	0.0003	0.000	0.000
Diallyl sulfide-D	Garlic	1.70	0.0003	0.089	0.28	0.0005	0.000	0.000	0.000
Dimethyl sulfide	Cabbage, Sulfur, Marine	1.66	0.000	0.000	0.002	0.000	0.000	0.000	0.000
Acetic acid-D	Spicy sour	1.53	0.0001	0.002	0.000	0.000	0.99	0.000	0.000
3-Methylbutanal	Chocolate, Green, Pungent	1.42	0.0033	0.000	0.000	0.0004	0.000	0.00017	0.000
Pentanal	Green grassy, faint banana, Pungent	1.14	0.0001	0.000	0.0002	0.000	0.028	0.0002	0.000
2-Butanone	Fruity, Camphor, Mint	1.08	0.045	0.99	0.002	0.059	0.001	0.00009	0.000

A: Odor descriptors from the database (http://www.thegoodscentscompany.com).

### 3.2 Analysis of volatile compounds in cigar tobacco leaves from different production areas using HS-SPME/SBSE-GC-O-MS

HS-SPME performs extraction by placing an adsorbent-coated fiber above the sample (headspace), primarily relying on volatile compounds diffusing from the sample matrix into the headspace before adsorbing onto the fiber. This method exhibits high extraction efficiency for volatile organic compounds (VOCs) such as aldehydes, ketones, alcohols, esters, and terpenes. Submerged-Bulk Solid-Phase Microextraction (SBSE) employs stirring bars coated with polydimethylsiloxane (PDMS) or other adsorbents immersed in the sample solution for extraction. As a liquid-phase extraction technique, it transfers analytes from the aqueous phase to the adsorbent via partitioning. Compared to HS-SPME, SBSE typically offers higher extraction capacity and is particularly suitable for medium-volatility and semi-volatile organic compounds (SVOCs), such as sesquiterpenes, long-chain fatty acid esters, and more complex heterocyclic compounds. Due to differences in extraction selectivity between HS-SPME and SBSE, these two methods are often considered complementary. By employing both techniques simultaneously, researchers can more comprehensively capture volatile compounds in cigar tobacco leaves, thereby obtaining a more complete aroma fingerprint.

**Figure 4:**
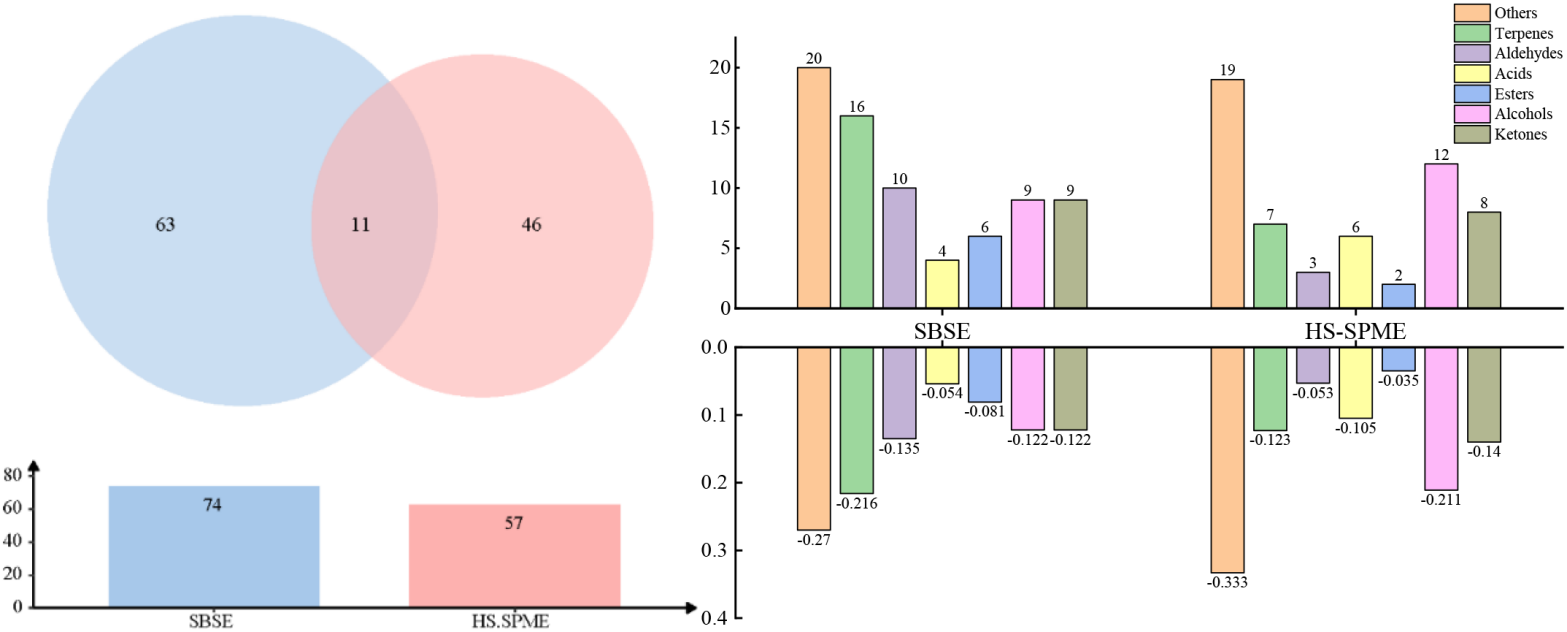
Distribution map of volatile compounds in cigar tobacco leaves under HS-SPME and SBSE treatment.

Pre-treatment techniques play a crucial role in the extraction and concentration of aromatic compounds in cigar tobacco leaves. Volatile compounds obtained using different extraction methods exhibit significant differences in both variety and concentration. As shown in the Venn diagram in **Figure 4**, a total of 120 compounds were identified (Yu et al., 2021; Zhou et al., 2024). Among these, HS-SPME and SBSE jointly detected 10 volatile substances, with 63 and 46 different types of aromatic compounds detected, respectively. The volatile compounds contained in cigar tobacco leaves are shown in **Table 4**. Interestingly, many aromatic compounds detected in cigar tobacco leaf aromas extracted using the SBSE method were not detectable by the SPME method. The results indicate that a single extraction method is insufficient for analyzing the profile of cigar tobacco leaves. Therefore, multiple pretreatment methods and GC-MS are required (Yao et al., 2021). SBSE, as an immersion enrichment extraction method characterized by simplicity, high sensitivity, good reproducibility, and low detection thresholds (Zhao C, et al., 2021), has been applied to some teas, such as roasted stem tea and Longjing tea, which are highly sensitive to volatile and semi-volatile flavors in liquid food materials (Wang M, et al., 2020, Diez-Simon et al., 2021).

**Table 4:** Identification of volatile components in cigar tobacco leaves based on HS-SPME and SBSE analysis methods.

Compounds	Name	RT	Fragrant melody		Growing region	Threshold	
			aroma description	OI		Air	Water
Acids							
A1	2-Aminophenacetic acid	10.462	Faint amine odor	1	INA, HB		
A2	cis-13-Octadecenoic acid	16.791	green	1	INA, HN		
A3	Acetic acid	17.77	Fruity, green, sweet	1	QL, INA, HB, YNmg, HN, SC	99.00	
A4	Propanoic acid	19.25	Sweet, fruity, apricot	1	QL, INA, HB, , HN, SC	2.19	
A5	Butanoic acid	20.72	Sweaty, acid, rancid	2	YNpr		
A6	Butanoic acid, 3-methyl-	21.37	Sweaty, acid, rancid	1	YNpr, SC	0.06	
A7	5-Methylhexanoic acid	22.94	–	–	QL	4.60	
A8	Pentanoic acid, 3-methyl-	22.96	Putrid cheese	2	QL, INA, YNpr	0.05	
A9	8-Hydroxy-2,2,8-trimethyldeca-5,9-dien-3-one	25.637	Fruity, oily richness	2	QL		
A10	Tetradecanoic acid	37.138	–	–	YNmg, YNpr , HN, HB, SC, QL, INA		10
Alcohols							
B1	trans-Farnesol	11.257	Floral, herbal	–	INA, YNmg, YNpr		
B2	Benzenemethanol, 2,4,5-trimethyl-	14.988	Woody, sweet	2	HN		
B3	cis-p-Mentha-2,8-dien-1-ol	16.465	–	–	INA, HB, SC, HN		
B4	2-Pentanone, 4-hydroxy-4-methyl-	16.49	–	–	QL	44.12	
B5	3,6,9,12-Tetraoxatetradecan-1-ol	19.81	–	–	QL		
B6	3,4-Dihydroxyphenylglycol, 4TMS derivative	20.11	–	–	QL		
B7	Hexaethylene glycol	23.38	–	–	QL		
B8	Geraniol	23.50	Sweet, floral, fruity, rose, waxy, citrus	3	YNmg, YNpr , HN, HB, SC, QL, INA	0.01	
B9	2,6-Octadien-1-ol, 3,7-dimethyl-	23.52	–	–	QL		
B10	1H-Benzocyclohepten-7-ol, 2,3,4,4a,5,6,7,8-octahydro-1,1,4a,7-tetramethyl-, cis-	25.139	Medicinal fragrance, ambergris	1	YNmg, HN, HB		
B11	2-[2-[2-[2-[2-[2-[2-(2-Hydroxyethoxy)ethoxy]ethoxy]ethoxy]ethoxy]ethoxy]ethoxy]ethanol	24.87	–	–	YNpr, YNmg, HN, HB, SC, QL, INA		
B12	2-Methyl-4-(2,6,6-trimethylcyclohex-1-enyl)but-2-en-1-ol	26.318	Woody, flaral, citrus	1	YNmg, HN, HB, SC, QL, INA		
B13	3-Pyridinemethanol, 4,5-dihydroxy-6-methyl-	27.766	Medicinal fragrance	1	YNmg, YNpr		
B14	1-Dodecanol, 3,7,11-trimethyl-	35.324	Sweet, woody	–	YNmg, YNpr, HB, QL, INA		
B15	3,7,11,15-Tetramethyl-2-hexadecen-1-ol	39.305	Green	–	HB, SC		
Aldehydes							
C1	Cyclopropanecarboxaldehyde, 2-methyl-2-(4-methyl-3-pentenyl)-, trans-(.+-.)-	8.288	Fruity Citrus Lemon	1	YNmg, YNpr, HB		
C2	Benzaldehyde	10.365	sweet, bitter, almond, cherry	–	YNmg, YNpr, INA, SC	0.085	0.75
C3	Nonanal	15.44	Citrus, Fatty	1-2	YNmg, YNpr, INA, HB	0.003	0.001
7C4	4-(2,2-Dimethyl-6-methylenecyclohexyl)butanal	15.738	Woody, citrus, resin	2	YNmg, YNpr, HB, HN		
C5	1-Cyclohexene-1-carboxaldehyde, 5,5-dimethyl-3-oxo-	16.968	Herbal fragrance, fresh	–	YNpr		
C6	1,3-Cyclohexadiene-1-carboxaldehyde, 2,6,6-trimethyl-	18.777	Woody, citrus	–	HB		
C7	Decanal	19.079	fresh, waxy	2	YNmg, YNpr , HB, SC	0.003	0.03
C8	1-Cyclohexene-1-carboxaldehyde, 2,6,6-trimethyl-	19.571	Fresh, fruity	1	YNpr, SC, HB, INA		
C9	4-(2,2-Dimethyl-6-methylenecyclohexyl)butanal	23.314	–	–	INA, HB		
C10	Phenylethyl Alcohol	24.3	Fruity, rose, sweet, apple	2-3	YNmg, YNpr , HN, HB, SC, QL, INA	0.14	
Ketones							
D1	5-Hepten-2-one, 6-methyl-	11.16	herbal, green, citrus, musty, lemon grass	1	INA, HB		
D2	3-Penten-2-one, 4-methyl-	12.28	Sweet, chemical	–	QL,HB,YNMG,YNPR	0.2	
D3	3-Hexen-2-one	13.8	–	–	HB		
D4	6-Methyl-5-heptadiene-2-one	15.515	Fruity, Green, woody	1	INA		0.1
D5	Ethanone, 1-(2-methylphenyl)-	18.484	sweet, hawthorn, powdery, anisic, coumarin, phenol, burnt, nutty, honey	1	YNPR,INA,SC		
D6	6-Methyl-5-heptadiene-2-one	20.45	Fruity, Green, woody	1	YNmg, YNpr , HN, HB, SC, QL, INA		0.1
D7	4,8-Dimethylnona-3,8-dien-2-one	21.3	Bergamot, cedarwood, rose	–	YNPR,INA		
D8	2-Undecanone, 6,10-dimethyl-	21.71	–	–	YNpr		
D9	2-Pyrrolidinone, 1-methyl-	21.95	–	–	QL,YNpr		
D10	Dehydromevalonic lactone	25.64	–	–	HB		
D11	2-Buten-1-one, 1-(2,6,6-trimethyl-1-cyclohexen-1-yl)-	26.278	Woody, fruity	1	QL,SC,HB		
D12	5,6-Dihydro-2(1H)-pyridinone	27.18	–	–	QL		
D13	5,9-Undecadien-2-one, 6,10-dimethyl-, (Z)-	27.228	Citrus, pine, rose	1	YNmg, YNpr , HN, HB, SC, QL, INA		
D14	Megastigmatrienone	31.508	–	–	YNmg, YNpr , HN, HB, SC, QL, INA		
D15	7-Isopropenyl-1,4a-dimethyl-4,4a,5,6,7,8-hexahydro-3H-naphthalen-2-one	37.528	Ambergris, Cedarwood	–	INA,SC		
D16	2-Pentadecanone, 6,10,14-trimethyl-	38.878	Oily, herbal, jasmin, celery, woody	–	QL		
Esters							
E1	Cyclohexene, 1-methyl-4-(1-methylethyl)-	12.545	Nutty	1	YNmg, YNpr , HN, HB, SC, QL, INA		
E2	Cyclohexanol, 1-methyl-4-(1-methylethenyl)-, acetate	13.077	Pine, citrus, herbal	1	YNpr , HN, HB, SC, QL, INA		
E3	Benzoic acid, 4-isopropenylcyclohexenylmethyl ester	17.706	Green	–	YNpr		
E4	Bicyclo[3.1.1]hept-2-en-6-ol, 2,7,7-trimethyl-, acetate, [1S-(1α,5α,6β)]-	18.364	Bitter (almond-like), nutty	2	YNmg, QL, HB		
E5	Ethyl 5-amino-1,2,3-thiadiazole-4-carboxylate	22.08	–	–	QL,HB		
E6	Acetic acid, 1-[2-(2,2,6-trimethyl-bicyclo[4.1.0]hept-1-yl)-ethyl]-vinyl ester	25.242	Fruity, freash	2	YNmg		
E7	Octaethylene glycol monododecyl ether	27.39	–	–	YNmg		
E8	Cyclopropaneoctanoic acid, 2-[(2-pentylcyclopropyl)methyl]-, methyl ester, trans,trans-	39.089	Resinous, smoky	–	YNmg, YNpr, QL		
Terpenes							
F1	1,3-Cycloheptadiene	4.832	–	–	YNmg, QL		
F2	3-Methylenecyclohexene	4.86	–	–	YNmg, HN, HB, INA		
F3	p-Xylene	7.378	strong, sweetish	–	YNmg, YNpr , HN, HB, SC, QL, INA	0.25	1
F4	Cyclohexene, 3-(1-methylethyl)-	7.71	Pine, citrus, spicy	–	YNmg, YNpr , HN, HB, SC, INA		
F5	Cyclohexene, 6-(2-butenyl)-1,5,5-trimethyl-, (E)-	9.289	Nutty	2	YNmg, YNpr, INA		
F6	Cyclohexene, 3-methyl-6-(1-methylethyl)-, trans-	9.878	Green (unripe/vegetal)	–	YNmg, YNpr , HN, HB, SC, QL, INA		
F7	Cyclohexene, 1-methyl-4-(1-methylethenyl)-, (S)-	10.268	Terpene, pine, herbal, peppery	1	HB, QL		0.034
F8	1,5,5-Trimethyl-6-methylene-cyclohexene	11.407	Citrus, woody	1	SC		
F9	Bicyclo[3.1.0]hex-2-ene, 2-methyl-5-(1-methylethyl)-	11.881	Pine, woody	1	YNmg, YNpr , HN, HB, SC, INA		
F10	D-Limonene	12.905	Citrus	2	YNmg, YNpr , HN, HB, SC, QL, INA		0.03
F11	Eucalyptol	13.69	Sweet, Mint	2-3	YNmg, YNpr , HN, HB, SC, QL, INA	0.02	
F12	1,5,9,11-Tridecatetraene, 12-methyl-, (E,E)-	13.781	Green (cucumber-like)	–	YNmg, YNpr		
F13	beta.-Ocimene	14.32	Apple, pear, fruity	1	YNmg, YNpr , HN, HB, SC, QL, INA	0.03	
F14	Styrene	14.36	Penetrating, balsamic, gasoline	–	YNmg, YNpr , HN, HB, SC, QL, INA	3	
F15	Caryophyllene oxide	17.992	Sweet, fresh, dry, woody, spicy	1	YNpr		0.41
F16	4-Nonene, 5-butyl-	25.494	Fat, grassy	1	QL, HN		
F17	n-Octylpentaoxyethylene	27.91	–		QL, INA		
F18	α-Dehydro-ar-himachalene	38.935	Woody, resinous	1	QL		
F19	Isolongifolene, 4,5,9,10-dehydro-	39.267	–	–	HN		
Others							
G1	Geranyl vinyl ether	11.189	Floral, herbal	–	YNmg, HN, HB, QL		
G2	1H-3a,7-Methanoazulene, octahydro-1,4,9,9-tetramethyl-	11.196	Nutty	1	YNmg		
G3	Bicyclo[3.1.1]heptane, 6,6-dimethyl-2-methylene-, (1S)-	11.996	Dry, woody, fresh, pine, Hay, green, resinous	1	QL		
G4	3-Hydroxy-N,N-dimethylpropanamide	12.76	–	–	HN, SC, INA		
G5	1-Isopropenyl-3-propenylcyclopentane	13.851	Woody, pine, citrus	1	HB, SC, INA		
G6	Pyridine, 2-methyl-	13.87	–	–	QL		
G7	Benzene, 4-ethyl-1,2-dimethyl-	14.39	–	–	QL	0.003	
G8	3-Hydroxy-N,N-dimethylpropanamide	14.82	–	–	HN, SC, INA		
G9	Benzenamine, 2-ethyl-6-methyl-	14.857	Nutty	2	YNmg, HN		
G10	Benzenamine, N-ethyl-3-methyl-	14.971	Nutty	2	YNmg, YNpr , HN, HB, SC, QL, INA		
G11	Benzene, 1-methyl-4-(1-methylethenyl)-	15.149	Phenol, spicy, clove, guaiacol	1	YNpr, INA		0.085
G12	3-Hydroxybenzoic acid, 2TMS derivative	15.257	Coffee, fatty, grassy	2	YNmg, YNpr , HN, HB, SC		
G13	N-(2-Ethylphenyl)-3-(isonicotinoylhydrazono)butyramide	15.864	Coffee	3	YNmg, YNpr		
G14	Ethanone, 1-(3-pyridinyl)-	15.91	Popcorn, tobacco, or roasted grains (coffee)	1	HN, INA		
G15	Pyridine, 3,5-dimethyl-	16.07	Sweet	1	INA		
G16	Pyrazine, 2-methoxy-3-(1-methylethyl)-	17.6	–	–	QL	0.0000039	
G17	Octadecane, 6-methyl-	19.177	–	–	YNmg, HB		
G18	Aromadendrene oxide-(2)	22.976	–	–	YNmg, YNpr , HN, HB		
G19	1,4-Bis(trimethylsilyl)benzene	22.891	Fruity, smoky, sweet, floral	1	YNpr		
G20	Hexaethylene glycol monododecyl ether	23.37	–	–	QL		
G21	Ethanone, 1-(4-pyridinyl)-	23.68	–	–	QL		
G22	Pyridine, 2-(1-methyl-2-pyrrolidinyl)-	24.06	–	–	YNmg, QL		
G23	Pyridine, 3-(1-methyl-2-pyrrolidinyl)-, (S)-	24.39	Burnt, smoky	3	YNmg, YNpr , HN, HB, SC, QL, INA		
G24	Caryophyllene oxide	26	Sweet, fresh, dry, woody, spicy	2	YNpr		0.41
G25	Pyridine, 3-(3,4-dihydro-2H-pyrrol-5-yl)-	26.747	–	–	YNmg, YNpr , HN, HB, SC, QL, INA		
G26	Myosmine	27.12	–	–	YNmg, YNpr , HN, HB, SC, QL, INA		
G27	Nicotyrine	28.17	–	–	YNmg, YNpr , HN, HB, SC, QL, INA		
G28	6-Quinolinamine, 2-methyl-	28.349	Ammoniacal	1	YNmg, YNpr , HB		
G29	2,3’-Dipyridyl	30.009	–	–	YNmg, YNpr , HN, HB, SC, QL, INA		
G30	Neophytadiene	38.237	Woody, Green	1-2	YNmg, YNpr , HN, HB, SC, QL, INA		

Odor Intensity (determined by GC-Olfactometry), OI: Abbreviation for “the intensity of fragrance perceived through an olfactometer.”

The influence of volatiles on flavor characteristic formation depends not only on their concentration levels but also on the odor threshold of the substance. Based on the odor contribution theory, to characterize the flavor profiles of different cigar tobacco leaves and identify the key odor compounds that distinguish their stylistic differences, the odor contribution of each volatile compound was calculated using concentration data combined with literature-reported odor threshold information (Odor threshold) (**Table 5**), and the compounds contributing most significantly to the aromatic characteristics of cigar tobacco leaves were screened out (ROAV ≥1; VIP ≥1, P ≤ 0.05) (Jeleń et al., 2013; Liu et al., 2018). These compounds, along with OI, are shown in **Table 4**. Eleven and seven substances were identified as aromatic active compounds via HS-SPME and SBSE, respectively. Aroma components in tobacco originate from the transformation of precursor molecules or are generated through chemical reactions (Chen J, et al., 2023). Based on their origin pathways, they can be systematically classified into five causative groups: chlorophyll degradation products, carotenoid degradation products, Maillard reaction products, phenylalanine degradation products, camphene degradation products, and labdane degradation products (Yu et al., 2021). Ketones are renowned for their key role in tobacco aroma, with 6-methyl-5-hepten-2-one and 4-methyl-3-pentene-2-one contributing a range of sweet, fruity, woody, and grassy aromas. Notably, 6-methyl-5-hepten-2-one is recognized for its fruity aroma, primarily produced through the oxidation of unsaturated fatty acids and the Maillard reaction (Zhang et al., 2022). Nonanal exhibits lipid and citrus aromas (Lee et al., 2014). Several alcohols have also been identified, primarily including geraniol and phenethyl alcohol, which can regulate the hygroscopicity and mouthfeel of tobacco products. Additionally, heterocyclic compounds, including pyrrole, pyridine, furan, and pyrazine, are known for their strong roasted, nutty, and caramel aromas (Xie et al., 2002).

**Table 5:** Identification of key aromatic active components in cigar tobacco leaves using gas chromatography-olfactometry technology.

No.	Compounds	Descriptors of the actual smell	SBSE			HS-SPME		
			ROAV	VIP	OI	ROAV	VIP	OI
1	Cyclohexene, 1-methyl-4-(1-methylethenyl)-, (S)-	Terpene, pine, herbal, peppery	1.63	1.11745	1	1.63		1
2	D-Limonene	Citrus, Woody	100	1.1824	2	1.33		2
3	Nonanal	Citrus, Fatty	35.4		2			1
4	3-Penten-2-one, 4-methyl-	Sweet, chemical				14.92		
5	Eucalyptol	Sweet, Mint				14.87		2-3
6	2,6-Lutidine	A pungent alkaline odor similar to pyridine				27.12		1
7	6-Methyl-5-heptadiene-2-one	Fruity, Green, woody				3.59		1
8	Pentanoic acid, 3-methyl-	Putrid cheese				24.61		2
9	Geraniol	Sweet, floral, fruity, rose, waxy, citrus				8.31		3
10	Phenylethyl Alcohol	Fruity, rose, sweet, apple				31.89		2-3
11	Pyridine, 3-(1-methyl-2-pyrrolidinyl)-, (S)-	Burnt, smoky		7.68365	3		3.23684	3
12	2,3’-Dipyridyl	–		1.94808		2		
13	6-Quinolinamine, 2-methyl-	Ammoniacal		1.51212	1			
14	Bicyclo[3.1.1]heptane, 6,6-dimethyl-2-methylene-, (1S)-	Dry, woody, fresh, pine, Hay, green, resinous		1.02458	1			
15	Neophytadiene	Woody, Green			2		1.76481	1

Odor Intensity (determined by GC-Olfactometry), OI: Abbreviation for “the intensity of fragrance perceived through an olfactometer.”

The aromatic profiles of cigar tobacco leaves have been widely studied both domestically and internationally (Barata et al., 2011; Zhao Y, et al., 2021; Zhen et al., 2020). Aromatic descriptions are categorized into fruit-like aromatic profiles, floral aromatic profiles, sweet aromatic profiles, green aromatic profiles, woody aromatic profiles, roasted aromatic profiles, waxy aroma, and tobacco aroma (Chen Q, et al., 2023; Ni et al., 2019; Wang, 2002; Shi and Liu, 1998). This study identified 11 primary aroma profiles, including ammoniacal, sweet, citrus, Woody, Rose, Fatty, Green grassy, Fruity, Camphor, Mint, Putrid cheese, and Tobacco-like aromas. The main aroma profiles consistent with GC-IMS measurements include ammoniacal, fatty (chocolate, fat), green grassy, fruity, and camphor. Compared to previous studies, additional aromas include ammonia, citrus, mint, and camphor. The formation of ammonia is associated with the fermentation and aging of cigar tobacco leaves (Shi et al., 2023); mint and camphor aromas are typically accompanied by woody scents and are primarily associated with terpenoid compounds. Compared to flue-cured tobacco aromas, additional aromas include ammonia, citrus, rotten cheese, mint, and camphor (Guan et al., 2023).

### 3.3 Analysis of changes in volatiles in cigar tobacco leaves from different production areas using an electronic nose

PCA, as a pattern recognition method, can reveal differences in data. The greater the total variance of PCA, the better it reflects the original data. Volatile aromatic components from cigar tobacco leaves of different regions were collected using an E-nose, and stable response values at 58, 59, and 60 seconds were used for PCA. Principal component analysis showed that the cumulative contribution rate of the first two principal components reached 87.8% (PC1 = 80.2%, PC2 = 7.6%, λ1/λ2 = 2 = 10.55). Based on the Kaiser criterion (λ > 1), these two components were retained. As shown in **Figure 1**, the samples from the six regions exhibited a clustered distribution in the PC1-PC2 space.

**Figure 5:**
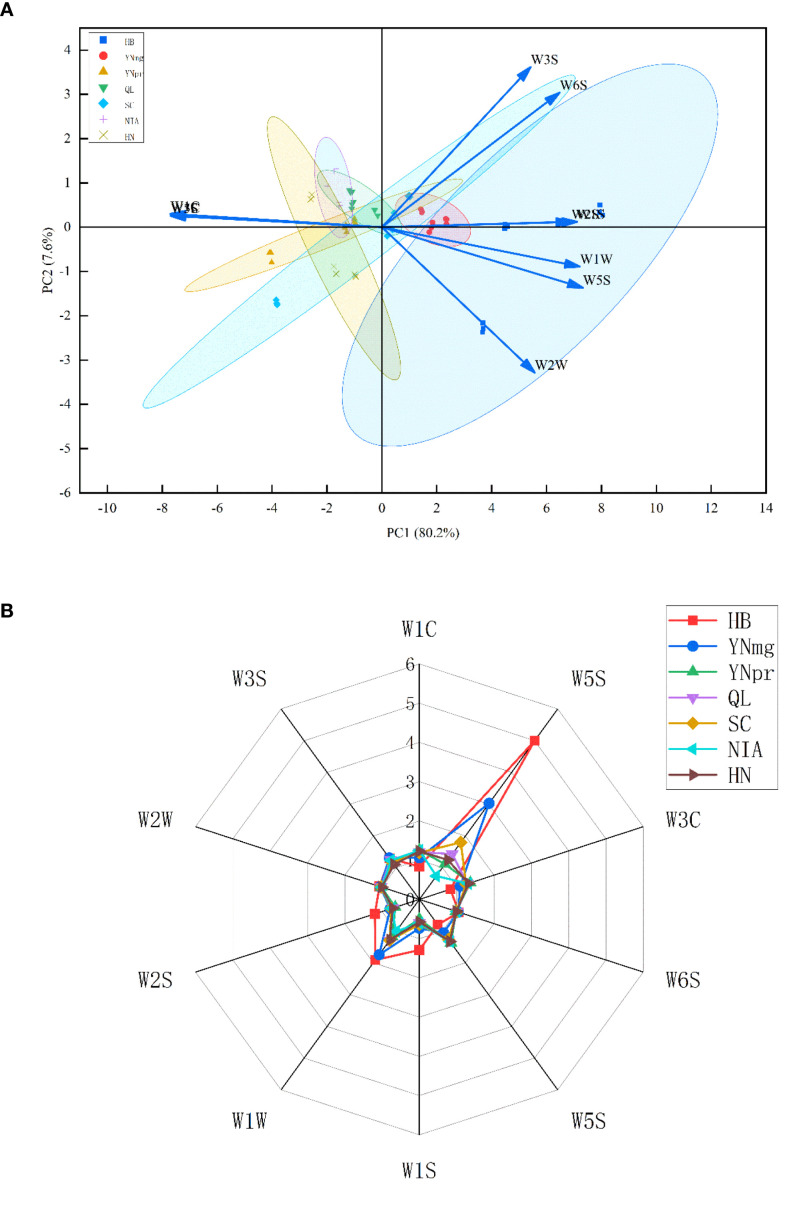
**(A)** Principal Component Analysis (PCA) Score Plot. The figure displays the scores of 10 metal oxide sensors (W1C, W5S, W3C, W6S, W1S, W1W, W2S, W2W, W3S) in a reduced-dimensional space defined by the first principal component (PC1, variance contribution rate 80.2%) and the second principal component (PC2, variance contribution rate 7.6%). The clustering and separation trends of different sensors on the score plot intuitively reflect their similarity and specificity in gas response characteristics. **(B)** Response Intensity Grid Map. This map visually displays the response intensity of each sensor to seven different samples (HB, YNmg, YNpr, QL, SC, NIA, HN) using color coding. Response values range from 0 to 6, revealing the sensitivity differences of the sensor array toward various samples.

As shown in **Figure 5**, the volatile aroma compounds extracted from each production area exhibited higher response values on the W5S (nitrogen oxide sensor) and W1C (sulfide sensor) compared to other sensors. The high sensor responses indicate that the cigar tobacco leaves contain elevated concentrations of nitrogen oxides and/or sulfide compounds. These substances may be responsible for olfactory characteristics in the cigar’s distinctive aroma profile, such as ammonia odor, strong pungent smell, spiciness, garlic odor, cabbage, sulfur, and gasoline notes(1). An electronic nose is an instrument that simulates the human olfactory system, performing pattern recognition of volatile compounds through a sensor array to provide a comprehensive fingerprint of volatile substances (Zhang et al., 2024). The high responsiveness of the W5S sensor is primarily attributed to the abundance of basic nitrogen-containing heterocyclic compounds in tobacco volatiles (Zhou et al., 2024). The nitrogen atoms in these molecules possess lone pair electrons, enabling strong adsorption on the sensor surface and potentially catalyzing oxidation reactions, resulting in significant changes in W5S conductivity (Zhou et al., 2024). These substances are key components of tobacco’s characteristic flavor and physiological activity. For instance, nicotine (Pyridine, 3-(1-methyl-2-pyrrolidinyl)-, (S)-) is a major alkaloid in tobacco, present in high concentrations and closely correlated with the response of the W5S sensor in the e-nose (Wu et al., 2024). The high response of W1C sensors primarily stems from the abundant reductive terpenes, alcohols, and aldehyde-ketone compounds in tobacco volatiles². The unsaturated bonds or functional groups like hydroxyl and carbonyl groups in these compounds exhibit strong reducing properties, undergoing oxidation reactions on the W1C sensor surface and causing increased conductivity. These volatiles contribute floral, fruity, and woody aroma characteristics to tobacco, serving as a key source of its flavor diversity (Jiang et al., 2024). For instance, D-limonene is often associated with citrus notes, eucalyptol imparts mint and camphor aromas, while geraniol contributes rose and floral scents. It is important to note that cross-sensitivity is also a significant consideration. Certain oxygen-containing compounds (e.g., aldehydes, alcohols) may elicit responses from both W1C and W5S, while some nitrogen-containing heterocyclic compounds may also produce signals on W1C due to their reductive groups. Consequently, the response of electronic nose sensors reflects the synergistic interaction of multiple volatile components in cigar tobacco, rather than the specific response to a single compound (Lin et al., 2025).

## 4 Conclusion

This study integrated multidimensional sensory chemistry techniques including GC-IMS, GC-MS/O, and electronic nose to systematically characterize the volatile aroma compound profile during the “cold aroma” stage of cigar tobacco leaves, identifying a total of 120 volatiles. Based on PCA and dual-indicator screening (ROAV/VIP), 15 compounds were identified as high-impact key aroma carriers. These primarily include Cyclohexene, 1-methyl-4-(1-methylethenyl)-, (S)-, D-Limonene, Nonanal, 3-Penten-2-one, 4-methyl-, Eucalyptol, 2,6-Lutidine, 6-Methyl-3,5-heptadiene-2-one, Pentanoic acid, 3-methyl-, Geraniol, Phenylethyl Alcohol, Pyridine, 3-(1-methyl-2-pyrrolidinyl)-, (S)-, 2,3’-Dipyridyl, 6-Quinolinamine, 2-methyl-, Bicyclo[3.1.1]heptane, 6,6-dimethyl-2-methylene-, (1S)-, Neophytadiene. Conferring a multifaceted aroma profile to cigars: ammoniacal, sweet, citrusy, woody, rose-like, fatty, green grassy, fruity, camphor, mint, putrid cheese, and tobacco-specific characteristics. Electronic nose responses further reveal characteristic zones for nitrogen oxides and sulfides (W5S/W1C) sensors.

It should be noted that the 60 °C headspace fingerprint only reveals the “cold aroma prototype” of unburned tobacco leaves and some thermal aroma precursors, without addressing subsequent transformations such as combustion, aging, and mouthfeel. While this cold aroma profile cannot directly predict smoking flavor, it serves as a targeted indicator for raw material selection and fermentation/aging process optimization, indirectly and directionally shaping the consumer-end aroma profile. Future work will integrate time-resolved pyrolysis, smoke analysis, and sensory omics to construct a comprehensive “cold aroma-hot aroma-aftertaste” prediction model, enabling precise design and iterative enhancement of cigar aroma quality.

## Data Availability

The datasets presented in this study can be found in online repositories. The names of the repository/repositories and accession number(s) can be found in the article/supplementary material.
